# Unsuccessful dispersal affects life history characteristics of natal populations: The role of dispersal related variation in vital rates

**DOI:** 10.1016/j.ecolmodel.2017.10.010

**Published:** 2017-12-24

**Authors:** Jacques A. Deere, Tim Coulson, Sarah Cubaynes, Isabel M. Smallegange

**Affiliations:** a Department of Zoology, University of Oxford, The Tinbergen Building, South Parks Road, Oxford, OX1 3PS, UK; b Institute for Biodiversity and Ecosystem Dynamics (IBED), University of Amsterdam, P.O. Box 94248, 1090 GE Amsterdam, The Netherlands

**Keywords:** Dispersal, Integral Projection Model, Deutonymph, Bulb mite, *Rhizoglyphus robini*

## Abstract

Individuals that disperse from one habitat to another has consequences for individual fitness, population dynamics and gene flow. The fitness benefits accrued in the new habitat are traded off against costs associated with dispersal. Most studies focus on costs at settlement and effects on settlement populations; the influence of dispersal to natal populations is assessed by monitoring change in numbers due to emigration. However, the extent to which natal populations are affected when individuals that invest in dispersal fail to disperse/emigrate is unclear. Here, we use an Integral Projection Model (IPM) to assess how developing into a disperser affects natal population structure and growth. We do so using the bulb mite (*Rhizoglyphus robini*) as a study system. Bulb mites, in unfavourable environments, develop into a dispersal (deutonymph) stage during ontogeny; these individuals are called dispersers with individuals not developing into this stage called non-dispersers. We varied disperser expression and parameterised IPMs to describe three simulations of successful and unsuccessful dispersal: (i) ‘no dispersal’ - dispersal stage is excluded and demographic data are from non-disperser individuals; (ii) ‘false dispersal’ - dispersal stage included and demographic data from non-disperser individuals are used; (iii) ‘true dispersal’ - dispersal stage included and demographic data are from individuals that go through the dispersal stage and from non-disperser individuals. We found that the type of dispersal simulation (no dispersal < false dispersal < true dispersal) and disperser expression increases generation time and reduces lifetime reproductive success and population growth rate. Our findings show that disperser individuals that fail to leave, can change the structure and growth of natal populations.

## Highlights

• Individual costs associated with dispersal morph expression carry over to negatively impact the natal population.
• Different dispersal scenarios and dispersal expression increase generation time but population growth rate declines.
• Increasing generation time is due to the addition of a life stage and to other life-history effects of the dispersal stage.
• We suggest that dispersing individuals that fail to disperse may affect the population dynamics of persistent natal populations.

## 1 Introduction

The importance of dispersal on the ecology of populations can be substantial (see Clobert et al., 2001). The role of dispersal into and out of populations impacts numbers, sex ratios, age structure, social dynamics and genetic structure of the population and so, as with reproduction and mortality, can be a crucial demographic process (Stenseth and Lidicker, 1992). With the dispersal process come associated costs that are traded off against (potential) fitness benefits that are accrued in the new habitat at both the individual and (meta-) population level (see review by Bowler and Benton 2005; Bonte et al., 2012). Dispersal is therefore a strategy that increases individual fitness in a heterogeneous (spatial and temporal) landscape by the process of moving the organism into a new environment, whereby variability in expected fitness between different habitat patches drives the evolution of dispersal (Bowler and Benton, 2005). Although there is some support for a purely genetic control of dispersal, there is widespread evidence that dispersal can be conditional upon a variety of traits (e.g. state-dependent, behavioural) and environmental conditions (McPeek and Holt, 1992, Clobert et al., 2004, Bowler and Benton, 2005).

Dispersal is a multi-phase life-history process that typically first entails energy investment into dispersal through increased reserves or into the expression of dispersal morphology (pre-departure), followed by the actual departure (initiating the eventual movement), transfer (the movement itself) and settlement (completion of the movement phase) (Bonte et al., 2012). Dispersal may fail during any of these stages, as individuals might not have sufficient reserves to invest into dispersal morphology, may be unable to find a host when dispersing through phoresy, could die during transfer or upon arriving at the settlement population, could fail to locate suitable habitat or disperse to suitable habitat and fail to find a mate (Bonte et al., 2012). Such unsuccessful dispersal could have important consequences for natal population and meta-population dynamics that can in turn feedback to influence dispersal expression and even its evolutionary trajectory. The study of unsuccessful dispersal has largely focused on the consequences of failure to disperse during the transfer stage and failing to establish during the settlement stage (Hanski, 1999, Bowler and Benton, 2005, Clobert et al., 2009, Burgess et al., 2012). To what extent pre-departure unsuccessful dispersal, where individuals have invested into dispersal but fail to leave their natal patch, affects natal populations in terms of population growth and structure is still unclear. Here, we examine the demographic consequences of the presence of unsuccessful dispersers, defined as individuals that have invested into dispersal morphology prior to intended departure but are not successful in leaving the natal population, on the size and structure of the natal population as well as key quantities that characterise population demography including population growth rate, mean lifetime reproductive success and generation time.

**Fig. 1:**
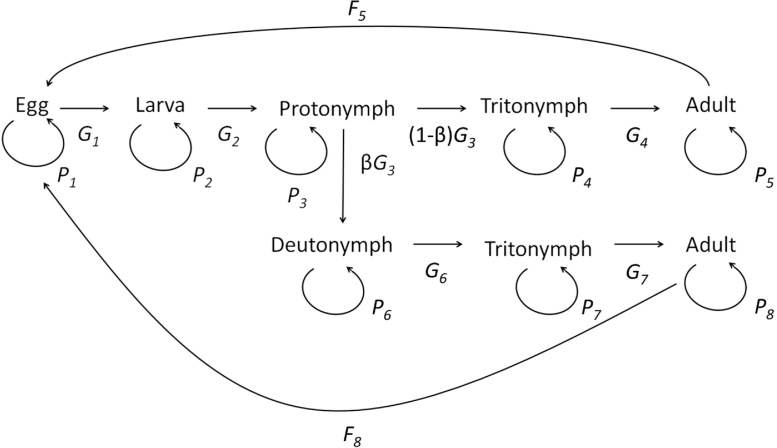
Life-cycle of the bulb mite, indicating the six life stages and the vital rates. From the life-cycle we calculated the survival (*P*) and fecundity (*F*) rates and the probability of surviving and growing into the next stage (*G*). Within the IPM *P*, *F* and *G* are represented as follows: *P* reflects the survival (*S*(*z,s,t*)), transition (*P*(*z,s,t*)) and growth, when staying in the same stage, (*G*(*z*’|*z,s,t*)) functions; *F* the reproduction (*R*(*z,s,t*)) and parent-offspring association (*D*(*z*’|*z,s,t*)) functions; and *G* the growth (*G*(*z*’|*z,s* *+* *1,t*)) function, after transitioning into the next stage (s indicates stage, *t* time, *z* body size and *z*’ body size at *t* + 1). The deutonymph stage is the facultative dispersal stage. See main text for details.

Expression of dispersal morphology prior to departure is often related to other individual characteristics such as energy reserves, mass or body size (Bonte et al., 2012, Deere et al., 2015). Different approaches exist to relate such characteristics of individuals to the dynamics of populations including Physiologically Structured Population Models (PSPMs) (Metz and Dieckmann, 1986), Matrix Population Models (MPMs) (Caswell 2001), Individual Based Models (IBMs) (Grimm and Railsback, 2005), and Integral Projection Models (IPMs) (Easterling et al., 2000). Of these, IPMs are useful for investigating simultaneous ecological and rapid evolutionary change in quantitative characters, life-history evolution and population dynamics (Easterling et al., 2000, Coulson, 2012, Merow et al., 2014). They are closely and easily linked to field and experimental data, can be applied to species with complex demography, and require relatively straightforward mathematical techniques from matrix calculus (Easterling et al., 2000, Ellner and Rees, 2006). Here we use IPMs to assess the consequences of unsuccessful dispersal by individuals during the pre-departure phase on natal population demography. As a study system we use the bulb mite (*Rhizoglyphus robini*, Acaridae). The bulb mite is an ideal study system for this research as its life-cycle contains a facultative, juvenile dispersal morph (deutonymph) (Fig. 1). Our IPM is density-independent, structured by life stage and body size (Fig. 1) and built from character-demography functions, which describe, for each life stage, the association between body size and female survival, growth and reproduction. We parameterise the IPM to describe three simulations of successful and unsuccessful dispersal: (i) ‘no dispersal’ - the dispersal stage is excluded and functions are parameterised using demographic data on non-disperser mites; (ii) ‘false dispersal’ - dispersal stage is included but functions are parameterised using demographic data on non-disperser mites; (iii) ‘true dispersal’ - dispersal stage is included and functions are parameterised using ‘true dispersal’ demographic data for those individuals that go through the dispersal stage and demographic data from non-disperser individuals. Comparing simulation (i) and (ii) allows us to assess the consequences for the natal population of adding a life stage to the life cycle, whereas the more interesting comparison between simulations (ii) and (iii) allows us to assess whether there are further demographic consequences of the dispersal stage, additional to that of adding an extra life stage. In our comparisons we analyse mean and variance in body size of each life stage, population growth rate (*λ_0_*), lifetime reproductive success (*R_0_*), and generation time. These results provide information on which demographic rates are most influential in natal population persistence under the different dispersal simulations.

## 2 Materials and methods

### 2.1 Study system and data collection

Bulb mites live in the soil and feed on bulbs and tubers and are pests of many crops and ornamentals (Diaz et al., 2000). Bulb mites are small (0.1–1.0 mm) and live for up to a few months (Diaz et al., 2000). From egg to adult, they go through a larval and two to three nymph stages, which take between 11 and 40 days depending on food quality (Smallegange 2011). Additionally, bulb mites have male dimorphism and males are either fighters, which kill other mites with their thickened third pair of legs, or scramblers, which do not have this modification and are defenceless (Radwan et al., 2000). Male morph is determined by the final instar (tritonymph) size, with larger tritonymphs developing into fighters. Dispersal occurs in both sexes via phoretic association with an arthropod host; attachment to the host is through the use of a sucker plate that is unique to the deutonymph stage (Diaz et al., 2000). Deutonymph expression is induced by unfavourable environmental conditions (e.g. low temperature, humidity, food quality) and is a non-feeding stage within the life-cycle (Diaz et al., 2000).

Data from Deere et al. (2015) were used to parameterise the IPMs. Briefly, in these experiments, first, females were isolated from stock cultures and allowed to lay eggs. The eggs were then reared individually to the adult stage and their life-history trajectory documented. Survival was scored on a daily basis for each individual. Surviving individuals were photographed daily, until maturation, using a Lumenera Infinity 3.1 camera (Lumenera Corporation, Ottawa, 22 Ontario, Canada) connected to a Meiji 20 EMZ-8TRD (10–45×) stereomicroscope and its length (body size) without mouthparts measured to the nearest 0.001 mm using Infinity Analyze Imaging Software (Lumenera Corp.). Mature females were mated with randomly chosen virgin males and eggs were counted and measured (measurements were limited to max. 10 eggs) on a daily basis until the female’s death. However, this initial dataset contained a very small proportion of individuals that expressed the deutonymph. We therefore isolated individual deutonymphs and protonymphs from the stock population to supplemented our dataset. By doing so we make two assumptions: (1) development during the egg to protonymph stages did not differ between individuals that did not develop into the deutonymph stage and those that did, and (ii) any differences in life-history traits between dispersers and non-dispersers are due to deutonymph expression and occur after this point in development. We were able to test the first assumption only. We compared the demographic trajectories of non-dispersing individuals (i.e. individuals that do not express the deutonymph stage) that were either isolated as eggs (initial dataset) or as protonymphs (supplemented data) and found no significant difference in growth and survival between non-dispersing individuals isolated from the stock culture as eggs or as protonymphs (see Deere et al., 2015). Given this we combined the initial and supplemented data.

### 2.2 Model construction and analyses

A number of steps were involved in model construction and analysis. First, we parameterised the character-demography functions that comprise each IPM for the three dispersal simulations (no dispersal, false dispersal, true dispersal) using bulb mite life-history data (see *2.2.1 Integral projection model* and Supplementary material) and, second, built an IPM for each dispersal simulation (see *2.2.1 Integral projection model*). Third, we varied the probability of transition into the dispersal stage for the false and true dispersal simulation to assess the effect of changes in dispersal expression on natal population demography (see *2.2.2 Dispersal expression*). All simulations were performed in R (version 3.0.2) (R Core Team, 2013).

#### 2.2.1 Integral projection model (IPM)

**Fig. 2:**
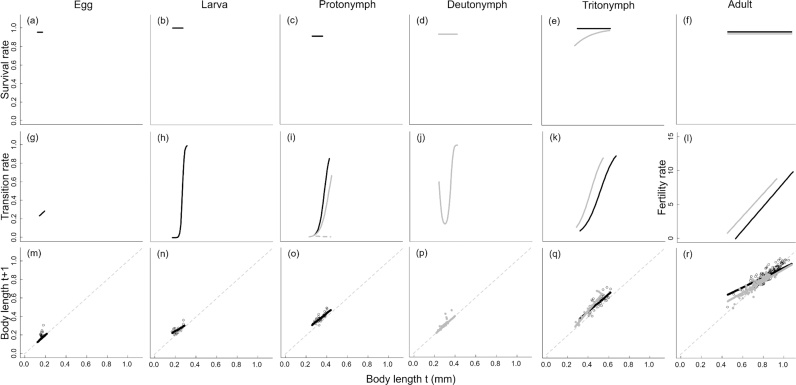
Character demography functions of each of the six life stages showing the relationship between body length and (a)–(f) survival rate, (g)–(k) transition rate (moving to next life stage), (l) fertility rate and (m)–(r) mean growth rate (when staying in same life stage) for the no dispersal simulation (black) and true dispersal simulation (grey). For the protonymph stage in the dispersal IPM, the transition function is a multinomial logistic and so has three probabilities. These probabilities are indicated by the solid grey line (probability of remaining in the protonymph stage), the dashed grey line (transition rate to the deutonymph stage), and the solid black line (transition rate to the tritonymph stage). The parent-offspring association function and growth when growing into the next life stage for both IPMs are not shown (parent-offspring association: egg size was not dependent on maternal size so mean and variance of egg size at *t* + 1 are constant; growth: growth between size *t* and *t* + 1 is described by growth rate of stage *s* + 1 as in this figure). Points are raw data.

The IPM is a kernel that describes how the body size *z* and stage *s* joint distribution at time *t* is influenced by survival, growth, reproduction and the parent-offspring association, resulting in a new body size *z*’ and stage *s*’ distribution at time *t* + 1:

(1) $$n_{t+1}\left(z^{\prime },s^{\prime }\right)=\overset{}{\underset{\Omega \times \text{s}}{\int }}K\left(z^{\prime },s^{\prime },z,s\right)n_{t}\left(z,s\right)dzds$$

where *z*’ indicates size at *t* + 1, *s*’ indicates stage at *t* + 1, and Ω designates the range of individual sizes. The character-demography functions from which the IPM kernel is built describe how body size *z* at each time *t* is related to: (1) the survival probability in stage *s* at time *t* + 1, *S*(*z,s,t*) (reflected in parameter ‘*P’* Fig. 1); (2) the transition probability that females develop into the next stage *s* at time *t* + 1, *P*(*z,s,t*) (reflected in parameter ‘*P’* Fig. 1); (3a) the increase in body size among survivors of size *z* that stay in stage *s* at time t + 1, *G*(*z*’|*z,s,t*) (reflected in parameter ‘*P’* Fig. 1); (3b) the increase in body size among survivors of size *z* that have moved to stage *s* + 1 at time *t* + 1, *G*(*z*’|*z,s* *+* *1,t*) (reflected in parameter ‘*G*’ Fig. 1); (4) the number of eggs produced by adult individuals of size *z* at time *t* + 1 (assuming a pre-breeding census), *R*(*z,s,t*) (reflected in parameter ‘*F*’ Fig. 1); and (5) the size of eggs, *z*’, produced by individuals of size *z* at time *t* + 1, *D*(*z*’|*z,s,t*) (reflected in parameter ‘*F*’ Fig. 1). Two of the functions, (3) and (5), also describe how the variance in size at time *t* *+* 1 is affected by size at time *t*. The IPM kernel comprises a number of equations based on the character demography functions and are listed in Table 1. Table 1, Eq. (2), provides a description of the dynamics of body size *z* (which is a continuous trait) for a population consisting of non-dispersers only (no dispersal simulation); Eq. (3) provides a description of a population of dispersers and non-dispersers (false and true dispersal simulation). In the no dispersal simulation, individuals transition from the protonymph directly to the tritonymph stage and then on to the adult stage (captured in the transition function *P*(*z,s,t*); see Eq. (2.2) Table 1), so that the number of life stages in the IPM is five (Fig. 1). The false and true dispersal IPMs also include the deutonymph stage, these IPMs then include individuals that go through the deutonymph stage and on to the tritonymph stage then to an adult, and those that do not enter the deutonymph stage and go through the tritonymph stage and on to an adult. In the false and true dispersal IPMs these two states of the tritonymph and adult stage are considered as different life stages and so the total life stages is eight (Fig. 1). Individuals that go through the deutonymph stage versus those that do not, differ in their survival and growth as tritonymphs and adults, and in their adult reproductive output (Deere et al., 2015). Character-demography functions of tritonymphs and adults in the two dispersal simulations are therefore split into two sets: one set for individuals that have not gone through the deutonymph stage and one set for individuals that have (Fig. 1). The difference between the false and true dispersal simulation is that survival, growth and reproduction of all life stages are parameterised as non-dispersers in the false dispersal IPM resulting in no difference in the life-histories of individuals. In the true dispersal IPM, disperser data are used to parameterise the survival, transition, growth and reproduction functions of tritonymphs and adults that went through the deutonymph stage in the true dispersal IPM (see Eqs. (3.1), (3.7)–(3.10) and (3.12) in Table 1), thus incorporating individuals with different life-histories. Since only adults reproduce, the *R*(*z,s,t*) and *D*(*z*’|*z,s,t*) functions are zero for all non-adult stages in all IPMs. Life-history data on female bulb mites were used to parameterise the IPM for the three different dispersal simulations (*2.1. Study system and data collection*). Parameter values used can be found in Table 2. These data showed significant differences in life-history traits between individuals that went through the deutonymph stage and individuals that did not (Deere et al., 2015). Details on how these functions were parameterized for each dispersal simulation (no dispersal, false dispersal and true dispersal) can be found in the Supplementary material.

**Table 1:** The Integral Projection Model (IPM) is a combination of the equations which generates a kernel which is approximated as a matrix. Equations are constructed from the five statistical demography functions: (1) Survival *S(z,s,t)*; (2) Transition *P(s|z,s,t)*; (3) Growth *G(z*’*|z,s,t)*; (4) Reproduction *R(z,s,t)* and (5) Parent-offspring association *D(z*’*|z,s,t)* (see main text for details). The equations calculate the number of females in each stage *s* at time *t* which is described by *n(z,s,t)* with the *R(z,s,t)* and *D(z*’*|z,s,t)* functions zero for all non-adult stages as only adults reproduce. Ω_s_ is a closed interval indicating the size domain of stage *s.*

	Life stage Equation	Description
Non-dispersal IPM		
(2.1)	$n\left(z^{'},1,t+1\right)=\int_{\Omega \text{s}}^{}D\left(z^{'}\text{|}z,s,t\right)R\left(z,s,t\right)n\left(z,s,t\right)dz\;\;\;\;s=5\;$	Egg production adults
(2.2)	$\begin{array}{c} n\left(z^{\prime },s+1,t+1\right)=\overset{}{\underset{\Omega \text{s}}{\int }}G\left(z^{\prime }\text{|}z,s+1,t\right)P\left(s+1\text{|}z,s,t\right)S\left(z,s+1,t\right)n\left(z,s,t\right)dz\\ n\left(z^{\prime },s,t+1\right)=\overset{}{\underset{\Omega \text{s}}{\int }}G\left(z^{\prime }\text{|}z,s,t\right)P\left(s\text{|}z,s,t\right)S\left(z,s,t\right)n\left(z,s,t\right)dz \end{array}\}1\leq s\geq 4$	Egg and all juvenile stages: staying in the current stage and developing into next stage
(2.3)	$n\left(z^{'},1,t+1\right)=\int_{\Omega \text{s}-1}^{}G\left(z^{'}\text{|}z,s,t\right)P\left(s\text{|}z,s-1,t\right)S\left(z,s-1,t\right)n\left(z,s-1,t\right)dz+\int_{\Omega \text{s}}^{}G\left(z\text{|}z,s,t\right)S\left(z,s,t\right)n\left(z,s,t\right)dz\;\;\;\;s=5\;\;$	Adults developing from Tritonymphs and surviving adults

Dispersal IPM		
(3.1)	$n(z^{'},1,t+1)=\int_{}D(z^{'}\text{|}z,5,t)R(z,5,t)n(z,5,t)dz+\int D(z^{'}\text{|}z,8,t)R(z,8,t)n(z,8,t)dz$	Egg production by non-dispersal and dispersal adults
(3.2)	$\begin{array}{c} n\left(z^{\prime },s+1,t+1\right)=\overset{}{\underset{\Omega \text{s}}{\int }}G\left(z^{\prime }\text{|}z,s+1,t\right)P\left(s+1\text{|}z,s,t\right)S\left(z,s,t\right)n\left(z,s,t\right)dz\\ n\left(z^{\prime },s,t+1\right)=\overset{}{\underset{\Omega \text{s}}{\int }}G\left(z^{\prime }\text{|}z,s,t\right)P\left(s\text{|}z,s,t\right)S\left(z,s,t\right)n\left(z,s,t\right)dz \end{array}\}1\leq s\geq 2$	Eggs and Larvae developing into the next stage and staying in the same stage
(3.3)	$n\left(z^{'},3,t+1\right)=\int_{}^{}G\left(z^{'}\text{|}z,3,t\right)P\left(3\text{|}z,3,t\right)S\left(z,3,t\right)n\left(z,3,t\right)dz\;$	Non-dispersal Protonymphs staying Protonymphs
(3.4)	$n\left(z^{'},6,t+1\right)=\int_{}^{}G\left(z^{'}\text{|}z,6,t\right)P\left(6\text{|}z,3,t\right)S\left(z,6,t\right)n\left(z,3,t\right)dz\;$	Deutonymphs developing from Protonymphs
(3.5)	$n\left(z^{'},4,t+1\right)=\int_{}^{}G\left(z^{'}\text{|}z,4,t\right)P\left(4\text{|}z,3,t\right)S\left(z,4,t\right)n\left(z,3,t\right)dz\;$	Non-dispersal Tritonymphs developing from Protonymphs
(3.6)	$n\left(z^{'},4,t+1\right)=\int_{}^{}G\left(z^{'}\text{|}z,4,t\right)P\left(4\text{|}z,4,t\right)S\left(z,4,t\right)n\left(z,4,t\right)dz\;$	Non-dispersal Tritonymphs staying Tritonymphs

(3.7)	$n\left(z^{'},6,t+1\right)=\int_{}^{}G\left(z^{'}\text{|}z,6,t\right)P\left(6\text{|}z,6,t\right)S\left(z,6,t\right)n\left(z,6,t\right)dz\;$	Deutonymphs staying Deutonymphs
(3.8)	$n\left(z^{'},7,t+1\right)=\int_{}^{}G\left(z^{'}\text{|}z,7,t\right)P\left(7\text{|}z,6,t\right)S\left(z,7,t\right)n\left(z,6,t\right)dz\;$	Dispersal Tritonymphs developing from Deutonymphs
(3.9)	$n\left(z^{'},8,t+1\right)=\int_{}^{}G\left(z^{'}\text{|}z,8,t\right)P\left(8\text{|}z,7,t\right)S\left(z,8,t\right)n\left(z,7,t\right)dz\;$	Dispersal adults developing from dispersal Tritonymphs
(3.10)	$n\left(z^{'},7,t+1\right)=\int_{}^{}G\left(z^{'}\text{|}z,7,t\right)P\left(7\text{|}z,7,t\right)S\left(z,7,t\right)n\left(z,7,t\right)dz\;$	Dispersal Tritonymph staying Tritonymphs
(3.11)	$n\left(z^{\prime },5,t+1\right)=\overset{}{\underset{}{\int }}G\left(z^{\prime }\text{|}z,5,t\right)P\left(5|z,5-1,t\right)S\left(z,5-1,t\right)n\left(z,5-1,t\right)dz+\overset{}{\underset{}{\int }}G\left(z\text{|}z,5,t\right)S\left(z,5,t\right)n\left(z,5,t\right)dz$	Non-dispersal adults developing from non-dispersal Tritonymphs and surviving non-dispersal adults
(3.12)	$n\left(z^{\prime },8,t+1\right)=\overset{}{\underset{}{\int }}G\left(z^{\prime }\text{|}z,8,t\right)P\left(8|z,8-1,t\right)S\left(z,8-1,t\right)n\left(z,8-1,t\right)dz+\overset{}{\underset{}{\int }}G\left(z\text{|}z,8,t\right)S\left(z,8,t\right)n\left(z,8,t\right)dz$	Dispersal adults developing from dispersal Tritonymphs and surviving dispersal adults

**Table 2:** Parameter estimates for each life stage from the bulb mite life-history data. Parameters are used to parameterize the functions for the non-dispersal and dispersal Integral Projection Models (IPMs). Details on how the functions were parameterised can be found in the supplementary material. Parameter *B* is body size at time *t*; N indicates sample size.

Life stage	IPM function	Function parameters values	N
*Non-dispersal IPM*			
Egg	Growth (staying in same stage)		65
	*Mean (mm)*	*y* = *B*	
	*Variance (mm^2^)*	*y* = 0.0001	
	Survival (fraction per day)	*y* = 0.956	297
	Transition (fraction per day)	$y=\frac{1}{1+\frac{1}{e^{\left(-1.437+8.647B\right)}}}$	97
	
Larvae	Growth (staying in same stage)		29
	*Mean (mm)*	*y* = 0.1174 + 0.6432*B*	
	*Variance (mm^2^)*	*y* = − 0.0008 + 0.0050*B*	
	Survival (fraction per day)	*y* = 0.999	112
	Transition (fraction per day)	$y=\frac{1}{1+\frac{1}{e^{\left(-6.933+29.429B\right)}}}$	47

Protonymph	Growth (staying in same stage)		39
	*Mean (mm)*	*y* = 0.0772 + 0.904*B*	
	*Variance (mm^2^)*	*y* = − 0.0007 + 0.0004*B*	
	Survival (fraction per day)	*y* = 0.909	166
	Transition (fraction per day)	$y=\frac{1}{1+\frac{1}{e^{\left(-14.3079+37.2537B\right)}}}$	108

Tritonymph	Growth (staying in same stage)		44
	*Mean (mm)*	*y* = 0.0776 + 0.9538*B*	
	*Variance (mm^2^)*	*y* = 0.0039 − 0.0042*B*	
	Survival (fraction per day)	*y* = 0.999	132

Tritonymph	Transition (fraction per day)	$y=\frac{1}{1+\frac{1}{e^{\left(-6.703+13.100B\right)}}}$	76

Adult	Growth (staying in same stage)		
	*Mean (mm)*	*y* = 0.3977 + 0.5359*B*	215
	*Variance (mm^2^)*	*y* = 0.0009 − 0.0004*B*	
	Survival (fraction per day)	*y* = 0.999	115
	Reproduction (no. per day)	y=0.5(−18.446+35.209B)	190
	Parent-offspring association (offspring-mother difference)		96
	*Mean (mm)*	*y* = 0.1638	
	*Variance (mm^2^)*	*y* = 0.00008	

*Dispersal IPM*			
Egg	Growth (staying in same stage)		65
	*Mean (mm)*	*y* = *B*	
	*Variance (mm^2^)*	*y* = 0.0001	
	Survival (fraction per day)	*y* = 0.956	297
	Transition (fraction per day)	$y=\frac{1}{1+\frac{1}{e^{\left(-1.437+8.647B\right)}}}$	97

Larvae	Growth (staying in same stage)		29
	*Mean (mm)*	*y* = 0.1174 + 0.6432*B*	
	*Variance (mm^2^)*	*y* = − 0.0008 + 0.0050*B*	
	Survival (fraction per day)	*y* = 0.999	112
	Transition (fraction per day)	$y=\frac{1}{1+\frac{1}{e^{\left(-6.933+29.429B\right)}}}$	47

Protonymph	Growth (staying in same stage)		39
	*Mean (mm)*	*y* = 0.0772 + 0.904*B*	
	*Variance (mm^2^)*	*y* = − 0.0007 + 0.0004*B*	
	Survival (fraction per day)	*y* = 0.909	166

Protonymph	Transition to deutonymph (fraction per day)	$y=\frac{1}{1+\frac{1}{e^{\left(-2.601+\left(-5.673\right)B\right)}}}$	137
	Transition to tritonymph (fraction per day)	$y=\frac{1}{1+\frac{1}{e^{\left(-11.220+26.235B\right)}}}$	137

Deutonymph	Growth (staying in same stage)		153
	*Mean (mm)*	*y* = *B*	
	*Variance (mm^2^)*	*y* = 0.0001	
	Survival (fraction per day)	*y* = 0.999	426
	Transition (fraction per day)	$y=\frac{1}{1+\frac{1}{e^{\left(55.05-385.54B+654.02B^{2}\right)}}}$	155

Tritonymph – T_P_	Growth (staying in same stage)		44
	*Mean (mm)*	*y* = 0.0776 + 0.9538*B*	
	*Variance (mm^2^)*	*y* = 0.0039 − 0.0042*B*	
	Survival (fraction per day)	*y* = 0.999	132
	Transition (fraction per day)	$y=\frac{1}{1+\frac{1}{e^{\left(-6.703+13.100B\right)}}}$	76

Tritonymph − T_D_	Growth (staying in same stage)		23
	*Mean (mm)*	*y* = − 0.0772 + 1.3570*B*	
	*Variance (mm^2^)*	*y* = − 0.0044 − 0.0060*B*	
	Survival (fraction per day)	$y=\frac{1}{1+\frac{1}{e^{\left(-0.4175+6.9435B\right)}}}$	119
	Transition (fraction per day)	$y=\frac{1}{1+\frac{1}{e^{\left(-6.275+14.933B\right)}}}$	45

Adult – A_P_	Growth (staying in same stage)		
	*Mean (mm)*	*y* = 0.3977 + 0.5359*B*	215
	*Variance (mm^2^)*	*y* = 0.0009 − 0.0004*B*	
	Survival (fraction per day)	*y* = 0.999	115

	Reproduction (no. per day)	y=0.5(−18.446+35.209B)	190
	Parent-offspring association (offspring-mother difference)		96
	*Mean (mm)*	*y* = 0.1638	
	*Variance (mm^2^)*	*y* = 0.00008	

Adult – A_D_	Growth (staying in same stage)		238
	*Mean (mm)*	*y* = 0.2816 + 0.6355*B*	
	*Variance (mm^2^)*	*y* = 0.0014 − 0.0016*B*	
	Survival (fraction per day)	*y* = 0.933	60
	Reproduction (no. per day)	y=0.5(−13.592+33.892B)	172
	Parent-offspring association (offspring-mother difference)		175
	*Mean (mm)*	*y* = 0.1689	
	*Variance (mm^2^)*	*y* = 0.0001	

T_P_ and A_P_ indicate tritonymphs and adults respectively that have not developed into the deutonymph stage during the life-cycle (developed directly from the protonymph stage during the life-cycle). T_D_ and A_D_ indicate tritonymphs and adults respectively that have developed into the deutonymph stage during the life-cycle (developed from the protonymph into the deutonymph during the life-cycle).

We used the IPMs to create predictions on population growth rate (*λ_0_*), lifetime reproductive success (*R_0_*) and generation time (*T*) for each dispersal simulation. To this end we discretised the IPM and divided the size domain of each stage into very small-width discrete bins (‘mesh points’; see Supplementary material for details). The population growth rate was calculated as the dominant eigenvalue from the matrix approximation of each IPM. Lifetime reproductive success was calculated from the *l_x_* and *m_x_* schedules, where *l_x_* is the survivorship function (probability of surviving from birth to age class *x*) and *m_x_* is the maternity function that describes reproduction (expressed as female offspring per female) (Stearns, 1992, Caswell, 2001). We calculated generation time as *T* = log(*R_0_*/log(λ_0_)); the time, *T*, required for the population to increase by a factor of *R_0_* (Caswell 2001).

#### 2.2.2 Dispersal expression

To assess the demographic consequences of an increase in dispersal expression (the proportion of deutonymphs in the population), the transition probability of developing from a protonymph to a deutonymph (deutonymph expression) in the false and true dispersal IPM was increased from 0.1 to 1.0 in increments of 0.1. For each transition probability, we calculated the population biology parameters (*λ_0_, T, R_0_*). Any change in these parameters across the range of transition probabilities will partly be due to the change in population structure (i.e. proportion of the population that become dispersers), which we can assess by comparing the no dispersal and false dispersal simulations, and partly due to the costs of investing in the dispersal stage (e.g. smaller size, increased age at maturity; see Table 3 and Deere et al., 2015), which we can assess by comparing the false and true dispersal simulations. This approach provides similar information to that of a life table response experiment (Caswell 2001).

**Table 3:** Population biology values generated from the IPMs (No dispersal IPM: Predicted; False Dispersal IPM: Predicted; True Dispersal IPM: Predicted) and from raw data (No dispersal IPM: Observed; False Dispersal IPM: Observed; True Dispersal IPM: Observed). Values generated were: population growth rate (*λ_0_*); generation time (*T*); lifetime reproductive success (*R_0_*); mean ± CI (*Z*)*^2^* of body size (E – eggs, L – larva, P – protonymph, D – deutonymph, TD – dispersal tritonymphs, TP – non-dispersal tritonymphs, AD – dispersal adults, AP – non-dispersal adults). NA – Not Available.

	No dispersal IPM		False Dispersal IPM		True Dispersal IPM	
Quantity	Predicted	Observed	Predicted	Observed	Predicted	Observed
*λ*_0_	1.32	NA	1.26	NA	1.26	NA
*R*_0_	76.62	67.08	57.71	NA	56.74	64.66^a^
*T*	15.51	14.21	17.58	NA	17.54	15.33
*Z_E_*	0.164 ± 0.002	0.164 ± 0.002	0.164 ± 0.004	0.164 ± 0.002	0.163 ± 0.003	0.164 ± 0.002
*Z_L_*	0.238 ± 0.007	0.256 ± 0.010	0.239 ± 0.007	0.256 ± 0.010	0.242 ± 0.007	0.256 ± 0.010
*Z_P_*	0.321 ± 0.018	0.383 ± 0.012	0.345 ± 0.016	0.383 ± 0.012	0.346 ± 0.015	0.383 ± 0.012
*Z_D_*	NA	NA	0.312 ± 0.016	0.299 ± 0.006	0.314 ± 0.018	0.299 ± 0.006
*Z_TP_*	0.441 ± 0.007	0.561 ± 0.021	0.476 ± 0.021	0.561 ± 0.021	0.478 ± 0.021	0.561 ± 0.021
*Z_TD_*	NA	NA	0.437 ± 0.027	NA	0.400 ± 0.032	0.464 ± 0.032
*Z_AP_*	0.775 ± 0.007	0.848 ± 0.023	0.788 ± 0.021	0.848 ± 0.023	0.788 ± 0.022	0.848 ± 0.023
*Z_AD_*	NA	NA	0.803 ± 0.024	NA	0.685 ± 0.030	0.766 ± 0.017

a Value is calculated as a ratio of lifetime reproduction of adults from deutonymph and adults not from deutonymph. Ratio is equivalent to the deutonymph transition rate of 0.02 (≈0.02:0.98; adults from deutonymph:adults not from deutonymph).

## 3 Results

### 3.1 Model performance

We compared predicted values of the population biology parameters for each of the three dispersal simulations to those derived from the data (with initial dispersal expression of 0.02). Generally there was a good match between the population biology values for the IPMs of the mite populations at equilibrium (Table 3).

In all three model simulations the predicted size for all stages, except the egg stage, was underestimated compared to observed values (Table 3). For the observed values, the disperser and non-disperser values did not differ for the egg, larvae and protonymph stages as the same data was used (see *2.1. Study system and data collection* and Deere et al., 2015). However, within each IPM simulation, for a given life stage the confidence intervals of the predicted size did not overlap with the confidence intervals of the predicted size of any other life stage, and predicted sizes followed the same level of size increase across life stages as observed values. We do advise some caution in the interpretation of the comparison of predicted values from the model and values derived from the data. The small sample size in combination with the number of life stages within the model precluded splitting the data into a training part to fit the model and testing part to validate the model which is necessary to provide a comprehensive independent comparison.

### 3.2 Effects on character demography functions

After parameterising the character-demography functions, the parameter estimates of the character-demography functions differed between dispersers and non-dispersers at the adult and tritonymph stage (Table 2, Fig. 2). Disperser tritonymphs transitioned at a smaller size than non-disperser tritonymphs resulting in smaller adult dispersers. Additionally, disperser tritonymphs had a lower survival rate at smaller and intermediate sizes than non-disperser tritonymphs. Adult fertility also differed between dispersers and non-dispersers. At smaller adult sizes, disperser adults had a higher fertility rate than non-dispersers, however the size range of reproducing females was larger for non-dispersers (Fig. 2). Additionally, the deutonymph transition rate resulted in a logit function with a significant quadratic term (body size^2^) (quadratic logit) (Fig. 2). However, it is more likely that larger individuals transition to the next stage, as they are probably older, and young individuals tend to be smaller and so have a lower probability of moving to the next stage (Caswell 2001). To reflect this we also used a linear logit function to describe this process to be able to generalise our results to other species with similar life histories. As such, we ran additional analyses but with the deutonymph transition rate fitted using a linear logit function (linear logit) (Supplementary material, Fig. A1).

### 3.3 Population level effects

**Fig. 3:**
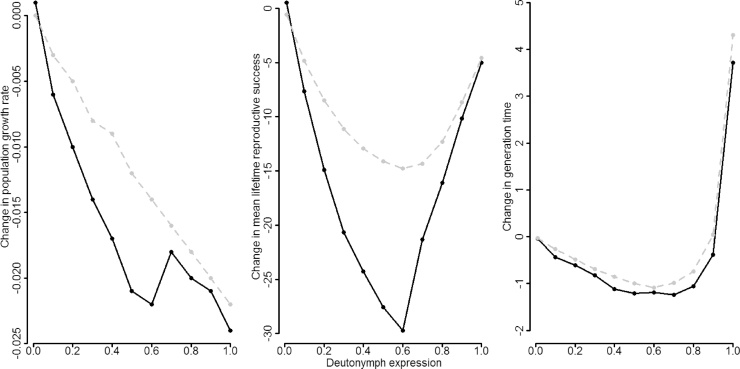
Difference (Δ) in population growth rate (λ_0_), mean lifetime reproductive success (*R*_0_) and generation time (*T*, days) with increasing deutonymph expression (deutonymph proportion: 0.1–1.0). The change is the difference between population biology values generated by the false and true dispersal IPMs (see text). Black lines and points based on a quadratic logit deutonymph transition function in the IPM, grey lines and points based on a linear logit deutonymph transition function in the IPM (see text for details).

Asymptotic population growth rate (*λ_0_*) and mean lifetime reproductive success (*R_0_*) are lower and generation time (*T*) higher in both the false and true dispersal simulations than in the non-dispersal simulation (Table 3). In the two dispersal simulations the initial dispersal expression in the model, based on parameterisation from the data, is <0.1 (0.02). In the case of the false dispersal simulation, *λ_0_* and *R_0_* are lower and *T* higher due to the additional life stage of a dispersing individual. In the true dispersal simulation the lower *λ_0_* and *R_0_* values and higher *T* are due to the combination of the reduced fertility and survival of dispersers, compared to non-dispersers, along with the additional life stage during disperser development. The false and true dispersal simulations reveal similar values; this is expected as the dispersal expression is low (0.02) and so fewer dispersers, with lower fertility and survival, contribute to the population. When increasing the dispersal expression in populations with dispersers, *λ_0_* is reduced further for the false and true dispersal simulations, as is *R_0_* for true dispersal (Table 4). However, in the case of *R_0_* for false dispersal, the values initially increase until deutonymph proportions of 0.6 and then decline. For *T*, the false and true dispersal simulations show similar patterns: initially there is a small increase, but when deutonymph proportions reach 0.6, there is a greater increase in *T* (Table 4). In order to show that the changes we see in *λ_0_*, *R_0_* and *T* are indeed due to investing in the deutonymph stage (true dispersal), and not solely due the extra time spent in the deutonymph life stage (false dispersal), we compared the difference-value of these two dispersal IPMs (see *2.2 Dispersal expression* above). Population biology values differed between the two dispersal IPMs (Table 4) with the *difference-value* becoming greater with increased expression of deutonymphs; negative values indicate a negative effect of deutonymph expression (Fig. 3, black lines). However, after deutonymphs reach proportions of 0.6, there is an opposite response in the difference-value for the *R_0_* and *T* values, where the difference-values become smaller (approach zero) between the two IPMs. *R_0_* in the true dispersal IPM parametrised with dispersers and non-dispersers declines steadily with increasing deutonymph proportion (Table 4). This is in contrast to *R_0_* in the false dispersal IPM parametrised with only non-dispersers which, initially, increases with deutonymph proportion but at proportions of 0.6 *R_0_* begins to decline (Table 4). In the case of *T*, the difference-value approaches zero at deutonymph proportions of 0.9 and then increases positively at a proportion of 1. In both dispersal IPMs, there is not much change in the value of *T* initially, but at deutonymph proportions of 0.6 *T* in both dispersal IPMs increases (Table 4). The same differences in population biology values between the false and true dispersal IPMs were calculated with IPMs which had a linear deutonymph transition function (Fig. 3, grey lines) (see *Effects on character demography functions* above – linear logit). This was done to test whether the results we found were not due to the non-linearity of the deutonymph transition function and how it affected the approximation of the IPM; an effect which may be specific to our study system. Results for the linear deutonymph transition function were similar with the exception of the change in *R_0_* which, although significant, was not as strong (Fig. 3, grey line).

**Table 4:** Population biology values generated from the false and true dispersal IPMs when increasing the proportion of deutonymphs in the model. Values generated were: population growth rate (*λ_0_*); generation time (*T*); lifetime reproductive success (*R_0_*).

	False Dispersal IPM			True Dispersal IPM		
Deutonymph proportion	*λ_0_*	*R_0_*	T	*λ_0_*	*R_0_*	T
Initial	1.259	58.00	17.58	1.259	58.52	17.54
0.1	1.262	61.14	17.70	1.256	53.48	17.26
0.2	1.263	64.00	17.81	1.253	49.09	17.20
0.3	1.264	65.95	17.91	1.250	45.29	17.09
0.4	1.263	67.21	18.00	1.246	42.94	17.02
0.5	1.263	67.88	18.09	1.242	40.31	17.00
0.6	1.260	67.63	18.24	1.238	37.92	17.05
0.7	1.221	52.25	19.79	1.203	30.91	18.55
0.8	1.192	39.95	21.00	1.172	23.86	19.94
0.9	1.150	26.23	23.33	1.129	16.06	22.95
1	1.093	14.86	30.30	1.069	9.825	34.02

Predicted mean body size differed between the three IPMs. Body sizes were larger for the larval and protonymph stages in the false and true dispersal simulations compared to the non-dispersal simulation (Table 3: significance was assessed by non-overlapping 95% confidence intervals). In the case of tritonymphs and adults, non-dispersers were larger (*Z_TP_, Z_AP_*) in the false and true dispersal IPMs compared to individuals in the non-dispersal IPM (*Z_T_,Z_A_*). Within the false and true dispersal IPMs, there were differences in mean size between tritonymphs and adults of non-dispersers (*Z_TP_*, *Z_AP_*) and dispersers (*Z_TD_*, *Z_AD_*); dispersers were smaller in both cases.

## 4 Discussion and conclusions

### 4.1 Individual and population-level consequences of unsuccessful dispersal: a comparison of dispersal simulations

Investment into reserves for dispersal or dispersal morphology likely also affect natal population characteristics if individuals are unable to disperse; yet we know of no study that has investigated this important aspect of dispersal ecology. Here, we used IPMs to investigate the demographic consequences for the natal population of such investments when individuals are unsuccessful in their efforts to disperse. Previously we found that for our study system, the bulb mite, investment into dispersal expression, by means of developing into the facultative deutonymph stage, is costly in terms of smaller body size at maturity and reduced fecundity (Deere et al., 2015). The results of the current study show that these costs carry over to affect individuals in their subsequent life stages. For example, in the true dispersal simulation, the simulation that incorporates differences in demographic rates between disperser individuals and individuals that do not disperse, dispersers in the population were on average smaller as a tritonymph and as an adult than non-dispersers (*Z_TP_, Z_AP_*, *Z_TD_, Z_AD_*; Table 3). This is not the case for the false dispersal simulation where only an additional life stage was included (CIs for mean body size overlap: *Z_TP_, Z_AP_*, *Z_TD_, Z_AD_*; Table 3).

Variation in body size can have far-reaching consequences for population structure. In our system, for example, tritonymph size is related to the expression of alternative reproductive tactics (Smallegange 2011). As in many other male dimorphic species (Taborsky et al., 2008), large male juveniles are more likely to develop into competitive ‘fighter’ males that aggressively defend their mates; whereas the smaller male juveniles are more likely to develop into the less competitive, ‘sneaker’ male that adopt a sneaker strategy to mate with females. We already know that such individual heterogeneity in alternative reproductive tactics can greatly affect the eco-evolutionary fluctuations of populations (Smallegange and Deere, 2014, Smallegange et al., 2017). The extent at which unsuccessful dispersal may play a role in this interaction remains to be investigated, particularly since dispersal expression and alternative reproductive tactic expression can be related (Deere, unpublished data). Finally, when comparing tritonymph and adult non-dispersers from the true dispersal simulation to the non-dispersal simulation, unexpectedly, the non-dispersers in the true dispersal simulation were larger. This is driven by the population structure and development rate of deutonymphs from protonymphs in the true dispersal simulation. Only protonymphs within a certain size range develop into deutonymphs; large protonymphs do not develop into deutonymphs (protonymph: min. length = 0.234 μm, max. length = 0.455 μm; deutonymph: min. length = 0.237 μm, max. length = 0.390 μm). This affects the average tritonymph (and ultimately adult) body size for non-dispersers as fewer, smaller non-dispersing individuals will develop into tritonymphs if there are deutonymphs in the population. Larger non-dispersing tritonymphs may also influence the proportion of scrambler males in the population as these tritonymph individuals are more likely to reach the critical size threshold to develop into fighters.

The results of this study also show that investment into (unsuccessful) dispersal can carry over to affect natal population characteristics. When looking at the population biology values we see a decrease in *λ_0_* and *R_0_* (no dispersal > false dispersal > true dispersal), and an increase in *T* (no dispersal < false dispersal < true dispersal) across the three dispersal simulations. The additional development time, because of the added dispersal life stage, and the fact that dispersers have lower growth, survival and fertility rates, ultimately results in a reduction in the number of recruits to the population, with recruits having lower fertility. Importantly, as these effects are seen even at a very low dispersal expression (0.02), this suggests that, even in relatively favourable environments where few individuals develop into a disperser, populations are negatively affected if dispersal morph expression is (partly) probabilistic.

### 4.2 .Effects of variation in dispersal expression

We next investigated the population-level consequences of an increase in dispersal expression. Comparing the changes in *λ_0_*, *R_0_* and *T* with increasing dispersal expression, between the false and true dispersal simulations, revealed that these changes were not solely due to the addition of an extra life stage. That is, the population-level consequences of an increase in dispersal expression differed between the false and true dispersal simulations. As dispersal expression increases, there is a larger difference of the *λ_0_*, *R_0_* and *T* values between the false and true dispersal simulations (Table 4; Fig. 3, difference-values (see *Methods*)). When dispersal expression equals ∼0.6, the response of *R_0_* and *T* change; at this point *R_0_* and *T* for the false and true dispersal simulations do not change in the same way (shape of the lines differ, Fig. 3). This is due to the response of the false dispersal IPM. In this simulation, *R_0_* initially increases before decreasing at a dispersal expression of ∼0.6 (Table 4). *R_0_* is determined by the *l_x_* and *m_x_* schedule and the change we see comes from a change in *l_x_* schedule with increasing dispersal expression; specifically in the egg, larva and protonymph stages (Fig. A2). With an initial increase in dispersal expression, the *l_x_* schedule shows an increase in the proportions of individuals in the egg, larva and protonymph stages. However, at a dispersal expression of ∼0.6 (and above), the proportion of individuals in these three stages starts decreasing. The change seen in generation time with increasing dispersal expression is also not intuitive. Initially *T* decreases with increasing dispersal expression, yet the expectation was that there would be an increase due to the negative effect on individuals expressing the dispersal stage (in addition to the time an extra stage brings). We do see the expected increase, but only at a proportion of 0.7. This outcome is likely due to the calculation of *T*. Generation time is calculated from *λ_0_* and *R_0_*, therefore the change in *T* is reflected by the response of *λ_0_* and *R_0_*. Here, *T* responds in a similar way to that of *R_0_* with increasing dispersal expression. *T* measures the weighted mean age of mothers, a measure that does not respond in the same way as our expectation of increasing *T* with increasing dispersal expression. An alternative measure of *T* may meet this expectation. One possibility is calculating cohort generation time (*T_c_*), this measure gives an indication of the average age of reproduction in a cohort (Tuljapurkar et al., 2009). The choice of parameter then becomes important in understanding the effect of increasing dispersal expression within a population and so there should be some caution when interpreting the values of *T* in this study. Nonetheless, we show that even when only considering an additional life stage (so excluding potential life-history costs of individuals; false dispersal simulation) the change to the population structure is more complex than just an increase in development time.

### 4.3 Applicability of results

Whether our results apply to other species depends firstly on the performance of our model, which was satisfactory in terms of estimated mean size of the life stages (importantly, mean sizes did not overlap) and population biology values and secondly, on how similar our character demography functions are to those of other species. As the deutonymph transition function in our model (henceforth “bulb mite IPM”) has an atypical hump-shape (Fig. 2), in order to have a realistic alternative for the transition function we removed the quadratic term of the function (Fig. A1) and re-ran the analysis (henceforth “general IPM”). The analysis of the “general IPM” resulted in population biology values similar to the “bulb mite IPM” (Fig. 3, grey lines). The similarity of the results, therefore, allow generalisation to other species with similar life-histories.

Unsuccessful dispersal from natal populations is highly likely to occur in phoretic species that depend on the presence of hosts to disperse, which involves a high degree of stochasticity. This host dependence can result in high variation in disperser numbers across space and time when host species fluctuate in presence and density. In species where dispersal is not host dependent, habitat fragmentation (e.g. reduced accessibility to habitat patches, Clobert et al., 2001) or environmental conditions (e.g. reduced migration levels during cloudy and rainy summers, Johnson, 1969) could affect the number of dispersers that ultimately remain in a natal population. We did not explore how costs of unsuccessful dispersal feeds back to affect individual size and deutonymph initiation. If dispersal in our study was density-dependent (and not determined by environmental quality), then potential feedbacks of the costs of unsuccessful dispersal can be included. This could be done using density-dependent IPMs where density is included as a term in the fundamental functions such that vital rates depend on population density. However, density-independent models can under certain circumstances provide an adequate approximation when density operates (see Caswell, 2001), therefore our simplified model can still provide insights into the consequences of unsuccessful dispersal on natal populations for the different life-histories that we studied here. Furthermore, the effect we see on natal populations may not necessarily be restricted to dispersal. Other forms of movement with distinct phenotypes, such as partial migration where a population contains both migratory and sedentary individuals (Kaitala et al., 1993, Chapman et al., 2011), may experience similar population level effects. In such a case, migratory and sedentary individuals often differ in traits other than migratory behaviour (e.g. size and dominance). These differences can lead to differences in vital rates which ultimately affect the population. However, having the morphological capability to migrate does not necessarily mean that an individual will migrate. In addition to the morphological component of migration (e.g. increased body size or wing development) a behavioural or physiological component (e.g. propensity to initiate migration or enzymes associated with locomotion ability) may be required to complete the migration event (Roff and Fairbairn 2001). Given this, it is feasible that individuals that initiate the migration process but do not migrate will ultimately affect the natal population with an effect similar to that in our study.

## 5 Outlook

Many models for dispersal have focused on either how resident populations are affected by dispersal rate (or distance) and ultimately metapopulation dynamics (Clobert et al., 2001, Hanski, 2001). This study focuses on the effects on the growth and structure of natal populations only; we see this as the first step of the effects of unsuccessful dispersal. The model presented here could form a basis for the structure of populations within a metapopulation model. The model is structured in a way that dispersal rates are calculated from only disperser individuals in the natal populations while also accounting for unsuccessful dispersers that remain in the natal habitat. Changes in population biology values will ultimately alter the dynamics of populations, whereby the magnitude of this change in dynamics depends on the level of dispersal expression (i.e. the distribution of the dispersal phenotype). Here we suggest that dispersing individuals that fail to disperse may affect the population dynamics of persistent natal populations, especially in fragmented landscapes. Indeed, conditions experienced in the natal habitat affect dispersal probability and, in turn, asymmetry between populations (Benard and McCauley, 2008). Consequently, how the dynamics of natal populations are affected could change the connectivity of a group of habitat patches, which has important implications for how connectivity is measured in natural populations. In a meta-population context, the contribution of dispersal to population persistence and synchrony will then be two-fold: (1) contribution of dispersers that disperse away from their natal population (Bowler and Benton, 2005) and (2) contribution of dispersers that fail to disperse and remain in their natal population (this study). When compared to how the contribution of dispersal is currently included in metapopulation models the approach presented here, where the dispersal contribution both in terms of successful dispersers and dispersers that fail to disperse, will change how individuals move between populations within these models.
