# Supplementary material for: Unsuccessful dispersal affects life history characteristics of natal populations: The role of dispersal related variation in vital rates
Source: Ecol Modell. 2017 Dec 24;366:37–47. doi: 10.1016/j.ecolmodel.2017.10.010 (PMC6472616; doi:10.1016/j.ecolmodel.2017.10.010)
Supplement: Supplementary file 1 [file mmc1.docx]

**Appendix A**

This online appendix accompanies the paper “Unsuccessful dispersal affects life history characteristics of natal populations: The role of dispersal related variation in vital rates” by J.A. Deere, T. Coulson, S. Cubaynes and I.M. Smallegange.

The first section describes the methods used to parameterize the character demography functions and the calculation of the mesh points used in the IPM. This is followed by two figures. Figure A1 shows the deutonymph transition rate used in the dispersal IPMs. Figure A2 shows the change in l_x_ schedule in the false dispersal simulation with increasing deutonymph proportions within the population.

***Parameter estimation***

Life-history data on female bulb mites taken from Deere et al. (2015) were used to parameterise the IPM (see main text for brief description of the data collection). We estimated the parameters using statistical models for the five character-demography functions: the survival function (1), transition function (2), growth function (3), reproduction function (4) and the parent-offspring association function (5) (Fig. 2 in main text).

*Non-dispersers*

Parameters were estimated following the method used by Smallegange et al. (2014). Function parameters were estimated using the following statistical models (summarized in Table A1): (1) Survival - generalised linear mixed model (GLMMs) with binomial error structure; (2) Transition rates – GLMM with binomial error structure; (3) Growth – GLMM with a Gaussian error structure; (4) Fertility rate (Reproduction) – GLMM with a Gaussian error structure; (5) Parent-offspring association – generalised linear model (GLM) with a Gaussian error structure. In all cases body length and body length squared were linear predictors and, with the exception of the parent-offspring association function where we fitted a GLM, mite identity was included as a random factor. The response variables for the five functions were: (1) Survival – from time *t* to time *t*+1 (this is binary and set as 0 or 1), (2) Transition - probability of growing to the next stage at time *t*+1, *γ_s,t+1_* (see below), (3) Growth - mean and variance in body size at time *t*+1, (4) Fertility - the number of eggs produced at time *t*+1, and (5) Parent-offspring association - mean and variance in size of eggs produced at time *t*+1 by each individual at time *t*. In the case of eggs their size at time *t*+1 equalled their size at time *t* as eggs do not increase in size.

During data collection it was not always possible to locate each individual every day. As such, the days where an individual was not seen but was still alive (i.e. observed alive the next day), body length was estimated (not including these observations would result in an underestimation of the survival function). The missing values were filled in by using the Gompertz function to estimate female body length at age *a* (Smallegange et al. 2014):

$z_{a}= z_{\infty}e^{{-e}^{-k(a-a_{0})}}$ (Eq. S1)

where *Z_a_* is body length (mm) at age *a* (days), *Z_∞_* is the mean maximum length (mm, at *a* = ∞), *k* is the instantaneous growth rate at age *a_0_* , and *a_0_* is the inflection point of the curve and the age at which absolute growth rate begins to decline.

In the case of function (2), we used duration in the life stage as an indicator of the transitioning to the next stage (Smallegange et al. 2014); i.e. time spent in the current stage depends on the probability of growing to the next stage (Caswell 2001). Therefore, *γ_s,t+1_* is given by *γ_s,t+1_* = 1/*d_s,t_*, where *d_s,t_* is the number of days that an individual still has to spend in stage *s*. This means that *d_s_* equals the total duration of stage *s* on the first day that a female is in stage *s*, and that when an individual develops from stage *s* into stage *s*+1 at time *t*+1, *d_s_*=1 so that *γ_s,t+1_* =1.

For function (3) and (5) the minimal model, which generated the predictors of mean size at time *t+*1, was utilised to generate the parameters for the variance around the mean size at *t*+1 by taking the squared residuals and fitting them against a statistical function of the same form as the mean size to estimate the variance in size at time *t+*1. The growth (3) and parent-offspring association (5) functions were then constructed using the equation following Easterling et al. (2000):

$y_{i}= \frac{1}{\sqrt{2\pi\sigma_{i}}}e^{\frac{{-(z^{'}- \mu_{i})}^{2}}{{2\sigma}_{i}^{2}}}$ (Eq. S2)

where *y*_i_ is either the growth or parent-offspring association function, *μ*_i_ describes the mean effect of the significant predictors on growth or parent-offspring association, and *σ*_i_ describes the squared residuals around *μ_i_*.

For details on construction of the IPMs and parameter values used see main text. Note that mite identity was included in the statistical analyses (except in the parent-offspring association function) but was not modelled within the IPMs.

*Dispersers*

In the case of dispersers the rates for eggs and larvae were the same as for the non-dispersers (see Fig. 2 in main text). However, in the disperser IPM, protonymphs can also develop into a deutonymph and we estimated the probability that a protonymph develops into a deutonymph, tritonymph or stays as a protonymph using a multinomial logisitic (which generated the three transition probabilities). For the multinomial logistic the linear predictor was body length at time *t*, the response variable was stage at time *t*+1 and the reference level was set as the protonymph stage. This gives the probability of developing into a tritonymph, a deutonymph or remaining as a protonymph as a function of individual size. As such, the regression coefficients $\beta\mu$ are the log of the ratio of the two probabilities of developing into a tritonymph or deutonymph over staying in the protonymph stage (the reference level/choice). For example, if $\beta\mu$ represents the effect of *µ* (size), we expect that for one unit change in *μ*, the relative risk of developing into a tritonymph over staying a protonymph will increase by $\exp(\beta\mu)$. The multinomial logistic analyses were performed in R (version 3.0.2) using the ‘mlogit’ package (R Development Core Team 2013). All other parameter estimates were calculated in the same way, and using the same analyses in R (version 3.0.2), as those for non-dispersing individuals.

In all statistical analyses a model simplification procedure was used. The full model was fitted, after which the least significant term from the highest order interaction downwards was identified and removed if the removal resulted in an insignificant increase in deviance. The full and reduced models are shown in Table A2. Significance of simplified models was assessed by performing a likelihood ratio test. The likelihood ratio (Λ) is calculated as Λ = 2(*LL_f_ – LL_j_*)/(*p_f_ – p_j_*); where *LL_i_* is the log-likelihood of the full model and *LL_j_* is the log-likelihood of the reduced model (*j* = r) or constant-only model (*j* = *c*). *p_i_* is the number of estimable parameters in the full model and *p_j_* is the number of estimable parameters in the reduced and constant-only model. The likelihood ratio is distributed, where $\nu$ is the difference in number of estimable parameters. The random factor was never removed during model simplification. Model assumptions and homoscedacity were confirmed by inspection of probability plots and error structures. All analyses were performed in R (version 3.0.2) with models fitted by maximum likelihood in the ‘lme4’ package (R Development Core Team 2013).

***Mesh point calculation***

Mesh points were created by dividing the size domain of each stage into very small-width discrete bins. A number of different bin sizes were used and results compared, this was done as an increase in the number of mesh points increases the numerical accuracy of the approximation (Ellner and Rees 2006). The body size domain of each stage was eventually divided into 50 size bins as a higher number of bins did not produce notably different results. Transition rates for the midpoint of two adjacent mesh points were estimated for each stage class. In the non-dispersal IPM, the final matrix size was 250X250 (50 bins x 5 stages = 250 mesh points); whereas in the dispersal IPM the final matrix size was 400X400 (50 bins x 8 stages = 400 mesh points). The dispersal IPM takes into account the different number of life stages of dispersers and non-dispersers as well two sets of tritonymph and adult life stages (tritonymphs and adults without dispersal stage, and tritonymphs and adults with dispersal stage) into a single IPM, hence there are eight stages in the final matrix and not five (see above; Fig. 1 in main text).

**Table A1.** Details of the statistical models used to calculate parameters of the functions used in the integral projection model. Statistical models are the full models and shown for all life stages for non-dispersers and dispersers. Full models were subject to model reduction; comparison of full and the final, reduced, model are shown in Table A2.

|  | **Model function** | **Statistical Model** | **Error structure** | **Model variables*** | |
| --- | --- | --- | --- | --- | --- |
| **Non-dispersers** | Growth | GLMM | Gaussian | *Egg* | Growth ~ 1 + (1|id) + block |
|  |  |  |  | *Larvae* | Growth ~ size_t + size_t^2 + block + (1|id) |
|  |  |  |  | *Protonymph* | Growth ~ size_t + size_t^2 + block + (1|id) |
|  |  |  |  | *Tritonymph* | Growth ~ size_t + size_t^2 + block + (1|id) |
|  |  |  |  | *Adult* | Growth ~ size_t + size_t^2 + block + (1|id) |
|  |  |  |  |  |  |
|  | Survival | GLMM | Binomial | *Egg* | Survival ~ size_t + size_t^2 + block + (1|id) |
|  |  |  |  | *Larvae* | Survival ~ size_t + size_t^2 + block + (1|id) |
|  |  |  |  | *Protonymph* | Survival ~ size_t + size_t^2 + block + (1|id) |
|  |  |  |  | *Tritonymph* | Survival ~ size_t + size_t^2 + block + (1|id) |
|  |  |  |  | *Adult* | Survival ~ size_t + size_t^2 + block + (1|id) |
|  |  |  |  |  |  |
|  | Transition | GLMM | Binomial | *Egg* | Duration ~ size_t + size_t^2 + block + (1|id) |
|  |  |  |  | *Larvae* | Duration ~ size_t + size_t^2 + block + (1|id) |
|  |  |  |  | *Protonymph* | Duration ~ size_t + size_t^2 + block + (1|id) |
|  |  |  |  | *Tritonymph* | Duration ~ size_t + size_t^2 + block + (1|id) |
|  |  |  |  |  |  |
|  | Reproduction | GLMM | Gaussian | *Adult* | Fertility ~ size_t + size_t^2 + block + (1|id) |
|  |  |  |  |  |  |
|  | Parent-offspring association | GLM | Gaussian | *Adult* | Egg size ~ size_t + size_t^2 + block |
|  |  |  |  |  |  |
|  |  |  |  |  |  |
| (Table A1 cont.) |  |  |  |  |  |
|  | **Model function** | **Statistical Model** | **Error structure** | **Model variables*** | |
| **Dispersers** | Growth | GLMM | Gaussian | *Egg* | Growth ~ 1 + (1|id) + block |
|  |  |  |  | *Larvae* | Growth ~ size_t + size_t^2 + block + (1|id) |
|  |  |  |  | *Protonymph* | Growth ~ size_t + size_t^2 + block + (1|id) |
|  |  |  |  | *Deutonymph* | Growth ~ 1 + (1|id) + block |
|  |  |  |  | *Tritonymph* | Growth ~ size_t + size_t^2 + block + (1|id) |
|  |  |  |  | *Adult* | Growth ~ size_t + size_t^2 + block + (1|id) |
|  |  |  |  |  |  |
|  | Survival | GLMM | Binomial | *Egg* | Survival ~ size_t + size_t^2 + block + (1|id) |
|  |  |  |  | *Larvae* | Survival ~ size_t + size_t^2 + block + (1|id) |
|  |  |  |  | *Protonymph* | Survival ~ size_t + size_t^2 + block + (1|id) |
|  |  |  |  | *Deutonymph* | Survival ~ size_t + size_t^2 + block + (1|id) |
|  |  |  |  | *Tritonymph* | Survival ~ size_t + size_t^2 + block + (1|id) |
|  |  |  |  | *Adult* | Survival ~ size_t + size_t^2 + block + (1|id) |
|  |  |  |  |  |  |
|  | Transition | GLMM | Binomial | *Egg* | Duration ~ size_t + size_t^2 + block + (1|id) |
|  |  |  |  | *Larvae* | Duration ~ size_t + size_t^2 + block + (1|id) |
|  |  |  |  | *Protonymph* | Duration ~ size_t + size_t^2 + block + (1|id) |
|  |  |  |  | *Deutonymph* | Duration ~ size_t + size_t^2 + block + (1|id) |
|  |  |  |  | *Tritonymph* | Duration ~ size_t + size_t^2 + block + (1|id) |
|  |  |  |  |  |  |
|  | Reproduction | GLMM | Gaussian | *Adult* | Fertility ~ size_t + size_t^2 + block + (1|id) |
|  |  |  |  |  |  |
|  | Parent-offspring association | GLM | Gaussian | *Adult* | Egg size ~ size_t + size_t^2 + block |
|  |  |  |  |  |  |
| *Predictor variable block refers to experimental blocks; id indicates identity of each individual; size_t is size of an individual at time *t*. Details of response and predictor variables can be found in the main text. | | | | | |

**Table A2.** Details of the full and final, reduced, models used to parameterize the integral projection model functions for each life stage. Statistical models for the egg, larvae and protonymph stages for non-dispersers and dispersers are the same. The deutonymph stage only occurs in dispersers.

| **Life stage** | | **IPM function** | | | **Statistical Model*** | | |
| --- | --- | --- | --- | --- | --- | --- | --- |
| Egg | | *Growth* | | |  | | |
|  | | Full model | | | y ~ 1 + (1|id) + treatment | | |
|  | | Final model | | | y ~ 1 + (1|id) | | |
|  | |  | | |  | | |
|  | | *Survival* | | |  | | |
|  | | Full model | | | y ~ size_t + size_t^2 + treatment + (1|id) | | |
|  | | Final model | | | y ~ 1 + (1|id) | | |
|  | |  | | |  | | |
|  | | *Transition* | | |  | | |
|  | | Full model | | | y ~ size_t + size_t^2 + treatment + (1|id) | | |
|  | | Final model | | | y ~ 1 + (1|id) | | |
|  | |  | | |  | | |
| Larvae | | *Growth* | | |  | | |
|  | | Full model | | | y ~ size_t + size_t^2 + treatment + (1|id) | | |
|  | | Final model | | | y ~ size_t + (1|id) | | |
|  | |  | | |  | | |
|  | | *Survival* | | |  | | |
|  | | Full model | | | y ~ size_t + size_t^2 + treatment + (1|id) | | |
|  | | Final model | | | y ~ 1 + (1|id) | | |
|  | |  | | |  | | |
|  | | *Transition* | | |  | | |
|  | | Full model | | | y ~ size_t + size_t^2 + treatment + (1|id) | | |
|  | | Final model | | | y ~ size_t + (1|id) | | |
|  | |  | | |  | | |
| Protonymph | | *Growth* | | |  | | |
|  | | Full model | | | y ~ size_t + size_t^2 + treatment + (1|id) | | |
|  | | Final model | | | y ~ size_t + (1|id) | | |
|  | |  | | |  | | |
|  | | *Survival* | | |  | | |
|  | | Full model | | | y ~ size_t + size_t^2 + treatment + (1|id) | | |
|  | | Final model | | | y ~ 1 + (1|id) | | |
|  | |  | | |  | | |
|  | | *Transition* | | |  | | |
|  | | Full model | | | y ~ size_t + size_t^2 + treatment + (1|id) | | |
|  | | Final model | | | y ~ size_t + (1|id) | | |
|  | |  | | |  | | |
|  | |  | | |  | | |
|  | |  | | |  | | |
| (Table A2 cont.) |  | | |  | |  |  | |  |
| **Life stage** | | | **IPM function** | | **Statistical Model*** | | |
| Deutonymph | | | *Growth* | |  | | |
|  | | | Full model | | y ~ 1 + (1|id) + treatment | | |
|  | | | Final model | | y ~ 1 + (1|id) | | |
|  | | |  | |  | | |
|  | | | *Survival* | |  | | |
|  | | | Full model | | y ~ size_t + size_t^2 + treatment + (1|id) | | |
|  | | | Final model | | y ~ 1 + (1|id) | | |
|  | | |  | |  | | |
|  | | | *Transition* | |  | | |
|  | | | Full model | | y ~ size_t + size_t^2 + treatment + (1|id) | | |
|  | | | Final model | | y ~ size_t + size_t^2 + (1|id) | | |
|  | | |  | |  | | |
| Tritonymph (non-disperser) | | | *Growth* | |  | | |
|  | | | Full model | | y ~ size_t + size_t^2 + treatment + (1|id) | | |
|  | | | Final model | | y ~ size_t + (1|id) | | |
|  | | |  | |  | | |
|  | | | *Survival* | |  | | |
|  | | | Full model | | y ~ size_t + size_t^2 + treatment + (1|id) | | |
|  | | | Final model | | y ~ 1 + (1|id) | | |
|  | | |  | |  | | |
|  | | | *Transition* | |  | | |
|  | | | Full model | | y ~ size_t + size_t^2 + treatment + (1|id) | | |
|  | | | Final model | | y ~ size_t + (1|id) | | |
|  | | |  | |  | | |
| Tritonymph (disperser) | | | *Growth* | |  | | |
|  | | | Full model | | y ~ size_t + size_t^2 + treatment + (1|id) | | |
|  | | | Final model | | y ~ size_t + (1|id) | | |
|  | | |  | |  | | |
|  | | | *Survival* | |  | | |
|  | | | Full model | | y ~ size_t + size_t^2 + treatment + (1|id) | | |
|  | | | Final model | | y ~ size_t + (1|id) | | |
|  | | |  | |  | | |
|  | | | *Transition* | |  | | |
|  | | | Full model | | y ~ size_t + size_t^2 + treatment + (1|id) | | |
|  | | | Final model | | y ~ size_t + (1|id) | | |
|  | | |  | |  | | |
| Adult (non-disperser) | | | *Growth* | |  | | |
|  | | | Full model | | y ~ size_t + size_t^2 + treatment + (1|id) | | |
|  | | | Final model | | y ~ size_t + (1|id) | | |
|  | | |  | |  | | |
|  | | | *Survival* | |  | | |
|  | | | Full model | | y ~ size_t + size_t^2 + treatment + (1|id) | | |
|  | | | Final model | | y ~ 1 + (1|id) | | |
|  | | |  | |  | | |
| (Table A2 cont.) |  | | |  | |  |  | |  |
| **Life stage** | | **IPM function** | | | **Statistical Model*** | | |
| Adult (non-disperser) | | *Reproduction* | | |  | | |
|  | | Full model | | | y ~ size_t + size_t^2 + treatment + (1|id) | | |
|  | | Final model | | | y ~ size_t + (1|id) | | |
|  | |  | | |  | | |
|  | | *Parent-offspring association* | | |  | | |
|  | | Full model | | | y ~ size_t + size_t^2 + treatment | | |
|  | | Final model | | | y ~ 1 | | |
|  | |  | | |  | | |
| Adult (disperser) | | *Growth* | | |  | | |
|  | | Full model | | | y ~ size_t + size_t^2 + treatment + (1|id) | | |
|  | | Final model | | | y ~ size_t + (1|id) | | |
|  | |  | | |  | | |
|  | | *Survival* | | |  | | |
|  | | Full model | | | y ~ size_t + size_t^2 + treatment + (1|id) | | |
|  | | Final model | | | y ~ 1 + (1|id) | | |
|  | |  | | |  | | |
|  | | *Reproduction* | | |  | | |
|  | | Full model | | | y ~ size_t + size_t^2 + treatment + (1|id) | | |
|  | | Final model | | | y ~ size_t + (1|id) | | |
|  | |  | | |  | | |
|  | | *Parent-offspring association* | | |  | | |
|  | | Full model | | | y ~ size_t + size_t^2 + treatment | | |
|  | | Final model | | | y ~ 1 | | |
|  | |  | | |  | | |
| *Statistical model used to generate parameters to parameterize the IPM functions | | | | | | | |

**
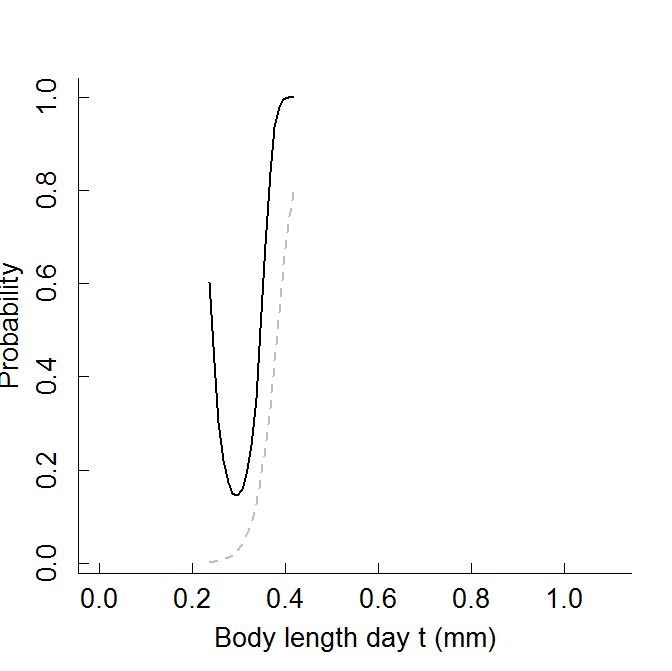
Supporting figures**

**Figure A1.** Deutonymph transition rate used in the dispersal IPM. Solid line indicates a logit function with a quadratic term, parameterised from dispersal life history data (quadratic logit transition function). Dashed line is a logit function without a quadratic term fitted to dispersal data which would be more representative of species with a similar life history to that of the bulb mite (linear logit transition function) (see main text for details).

**
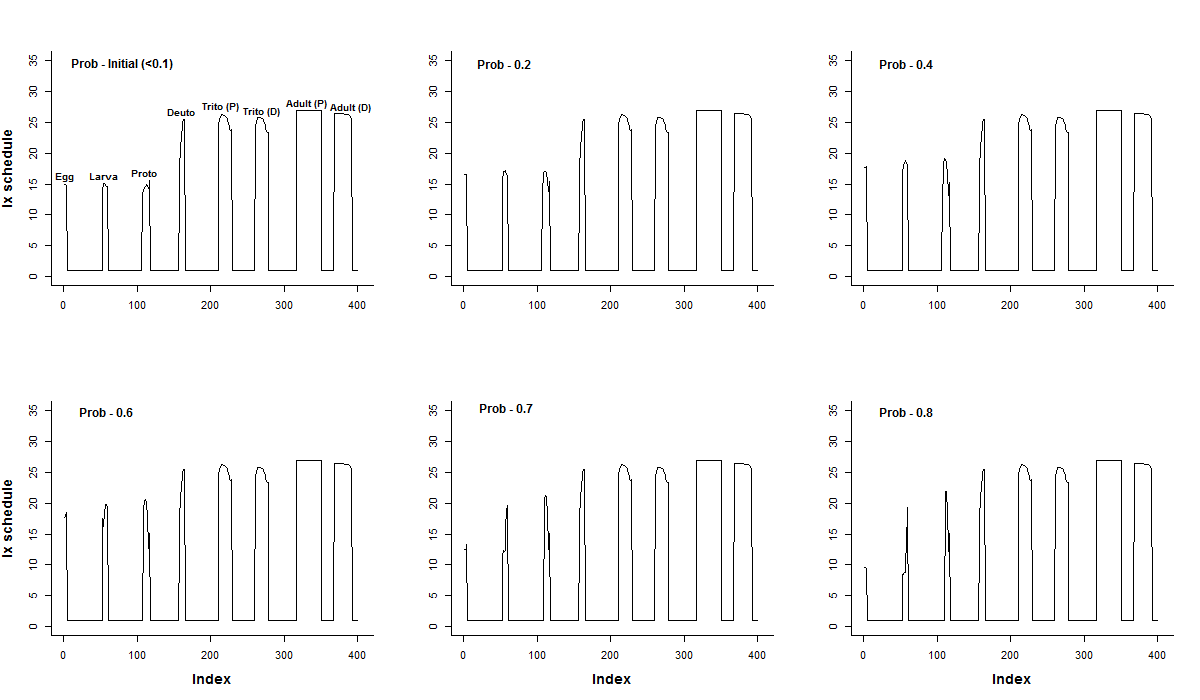
**

**Figure A2.** False dispersal simulation l_x_ schedule indicating each life stage at increasing proportions of dispersers (deutonymphs) within the population. The l_x_ schedule tracks a cohort from birth to death recording the number of individuals in each stage class. Index represents all life stages. Each life stage occurs at intervals of 50, with the each interval describing a size distribution from 0.2mm – 1.0mm (Egg – Index 1:50; Larva – Index 51-100; etc.). Life stages are labeled in the top left graph and are the same across all graphs. Deutonymph is the dispersal stage; TP – tritonymphs that developed from protonymphs; TD – tritonymphs that developed from deutonymphs; AP – adults that did not have a deutonymph stage during development; AD – adults that went through a deutonymph stage during development (see Fig.1 in main text).

**Literature cited in the Appendix**

Caswell, H. 2001. Matrix population models: Construction, analysis, and interpretation. Sunderland: Sinauer Associates, Massachusetts.

Deere, J. A., T. Coulson, and I. M. Smallegange. 2015. Life History Consequences of the Facultative Expression of a Dispersal Life Stage in the Phoretic Bulb Mite (*Rhizoglyphus robini*). PLoS ONE 10:e0136872.

Easterling, M.R., S.P. Ellner and P.M. Dixon. 2000. Size-specific sensitivity: Applying a new structured population model. Ecology 81: 694-708.

Ellner, S.P. and M. Rees. 2006. Integral Projection Models for Species with Complex Demography. The American Naturalist 167: 410-428.

R Core Team (2013). R: A language and environment for statistical computing. R Foundation for Statistical Computing, Vienna, Austria. URL http://www.R-project.org/.

Smallegange, I.M., J.A. Deere and T. Coulson. 2014. Correlative changes in life-history variables in response to environmental change in a model organism. American Naturalist 183: 784-797.
